# Analysis of imaging biomarkers and retinal nerve fiber layer thickness in *RPGR*-associated retinitis pigmentosa

**DOI:** 10.1007/s00417-021-05233-w

**Published:** 2021-07-21

**Authors:** Theresa H. Birtel, Johannes Birtel, Kristina Hess, Amelie C. Clemens, Moritz Lindner, Philipp Herrmann, Frank G. Holz, Martin Gliem

**Affiliations:** 1 Department of Ophthalmology, University Hospital Bonn, Bonn, Germany; 2 Center for Rare Diseases Bonn (ZSEB), University Hospital Bonn, Bonn, Germany; 3 Oxford Eye Hospital, Oxford University Hospitals NHS Foundation Trust, John Radcliffe Hospital, Oxford, UK; 4 Nuffield Department of Clinical Neurosciences, Nuffield Laboratory of Ophthalmology, University of Oxford, Oxford, UK; 5 Department of Neurophysiology, Institute of Physiology and Pathophysiology, Philipps University, Marburg, Germany

**Keywords:** Retinitis pigmentosa, Fundus autofluorescence, Optical coherence tomography, RNFL, Biomarker, Gene therapy

## Abstract

**Purpose:**

To investigate multimodal retinal imaging characteristics including the retinal nerve fiber layer (RNFL) thickness in patients with *RPGR*-associated retinitis pigmentosa (RP).

**Methods:**

This cross-sectional case–control study included 17 consecutive patients (median age, 21 years) with *RPGR*-associated RP who underwent retinal imaging including optical coherence tomography (OCT), short-wavelength fundus autofluorescence (AF) imaging, and RNFL scans centered on the optic disc. RNFL thickness was manually segmented and compared to clinical and imaging parameters including the transfoveal ellipsoid zone (EZ) width, the horizontal diameter of the macular hyperautofluorescent ring. RNFL thickness was compared to 17 age- and sex-matched controls.

**Results:**

In patients with *RPGR*-associated RP, the EZ width (*R*^2^ = 0.65), the central hyperautofluorescent ring on AF images (*R*^2^ = 0.72), and visual acuity (*R*^2^ = 0.68) were negatively correlated with age. In comparison to controls, a significantly (*p* < 0.0001) increased global RNFL thickness was identified in *RPGR*-associated RP, which was, however, less pronounced in progressed disease as indicated by the EZ width or the diameter of the central hyperautofluorescent ring.

**Conclusions:**

This study describes retinal characteristics in patients with *RPGR*-associated RP including a pronounced peripapillary RNFL thickness compared to healthy controls. These results contribute to the knowledge about imaging biomarkers in RP, which might be of interest for therapeutic approaches such as gene replacement therapies. 
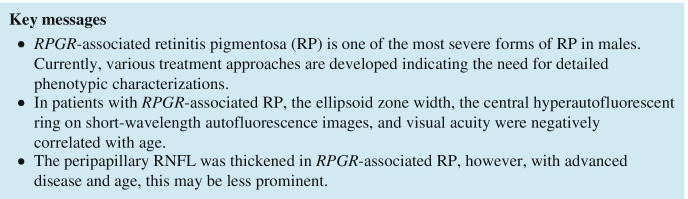

**Supplementary Information:**

The online version contains supplementary material available at 10.1007/s00417-021-05233-w.

## Introduction

Mutations in the *RPGR* gene are the major cause of X-linked retinitis pigmentosa (RP). The retinal phenotype is characterized by pronounced alterations with blindness often within the third or fourth decade of life. Thus, it represents one of the most severe forms of RP in males [1–9]. Female carriers might also be affected to a variable degree, for instance, due to skewed X-inactivation, and *RPGR* variants may also be found in sporadic cases [9–14]. Therefore, it appears crucial to screen families with a provisional diagnosis of autosomal dominant or sporadic inheritance for variants in X-linked genes as well [15, 16].

With the development of novel treatment approaches for *RPGR*-associated RP, in particular gene replacement therapy, an explicit disease characterization including multimodal retinal imaging is increasingly important [17–21]. Previous natural history studies have shown an exponential decline of the ellipsoid zone (EZ) width and the hyperautofluorescent ring [22–24].

A phenomenon not fully understood is inner retinal thickening observed in *RPGR*-associated RP [25]. Explanations for this phenomenon have been brought forward including that it represents a neuronal-glial remodeling associated with photoreceptor stress or loss, which has been observed in rodent models of retinal degeneration [25–27]. The peripapillary retinal nerve fiber layer (RNFL) thickness represents a marker for inner retinal thickness, which can be measured by optical coherence tomography (OCT) imaging. Previously, an abnormal RNFL thinning and thickening has been reported in RP [28–34]. Even though the integrity of the inner layer is crucial for patients qualifying for e.g. gene replacement therapies, retinal implants, or optogenetic approaches, no study has specifically investigated RNFL alterations depended on the genetic disease cause and its impact is not comprehensively understood.

The present study provides a phenotypic characterization of patients with *RPGR*-associated RP, quantifies retinal layers with a focus on RNFL thickness, and compares RNFL alterations to healthy controls.

## Methods

### Patients

The subjects included in this cross-sectional case–control study were identified at the Department of Ophthalmology, University of Bonn, a dedicated clinic for retinal dystrophies. The study was in adherence to the declaration of Helsinki. Institutional review board approval (Ethikkommission, Medizinische Fakultät der Rheinischen Friedrich-Wilhelms-Universität Bonn), and patients’ informed consent were obtained.

The clinical diagnosis of RP was based on the patient’s history, clinical examination, retinal imaging, and electrophysiologic assessment by full-field electroretinography (ERG). Genetic testing was performed as described previously [11, 35]. Exclusion criteria were any other pathology of the posterior pole unrelated to RP, any pathology affecting the ocular media like corneal opacities, cataract unusual for age or vitreous opacities, and highly unstable fixation, preventing adequate image acquisition. Furthermore, patients without RNFL imaging were excluded.

The RNFL thickness among the included patients was compared with a cohort of healthy controls (*n* = 17). These age-matched male subjects were unaffected by ophthalmic diseases and had a best-corrected visual acuity (BCVA) of 20/20 or better. Further cohort characteristics are provided in Supplementary Tables 1, 2.

### Clinical examination, image acquisition, and analysis

A complete ophthalmologic examination including BCVA testing, standardized slit-lamp examination, and dilated fundus examination was performed. Retinal imaging included fundus photography (Zeiss, Visucam, Oberkochen, Germany), wide-field pseudo-color- and AF fundus imaging (Optos PLC, Dunfermline, United Kingdom), spectral-domain optical coherence tomography (OCT) including RNFL ring scans (diameter of about 3.5–3.6 mm [36]) centered on the optic disc, and fundus autofluorescence (AF) imaging (both Spectralis HRA + OCT, Heidelberg Engineering, Heidelberg, Germany).

Macular hyperautofluorescent rings identified by AF imaging, using a 488 nm laser excitation light, were measured manually by outlining horizontal borders with the Heidelberg Eye Explorer Software (HEYEX, Heidelberg Engineering). Furthermore, the software was used to measure the extent of the foveal EZ (based on central foveal scan) on OCT imaging. Exemplary AF and corresponding OCT images are provided in Supplementary Fig. 1.

**Fig. 1:**
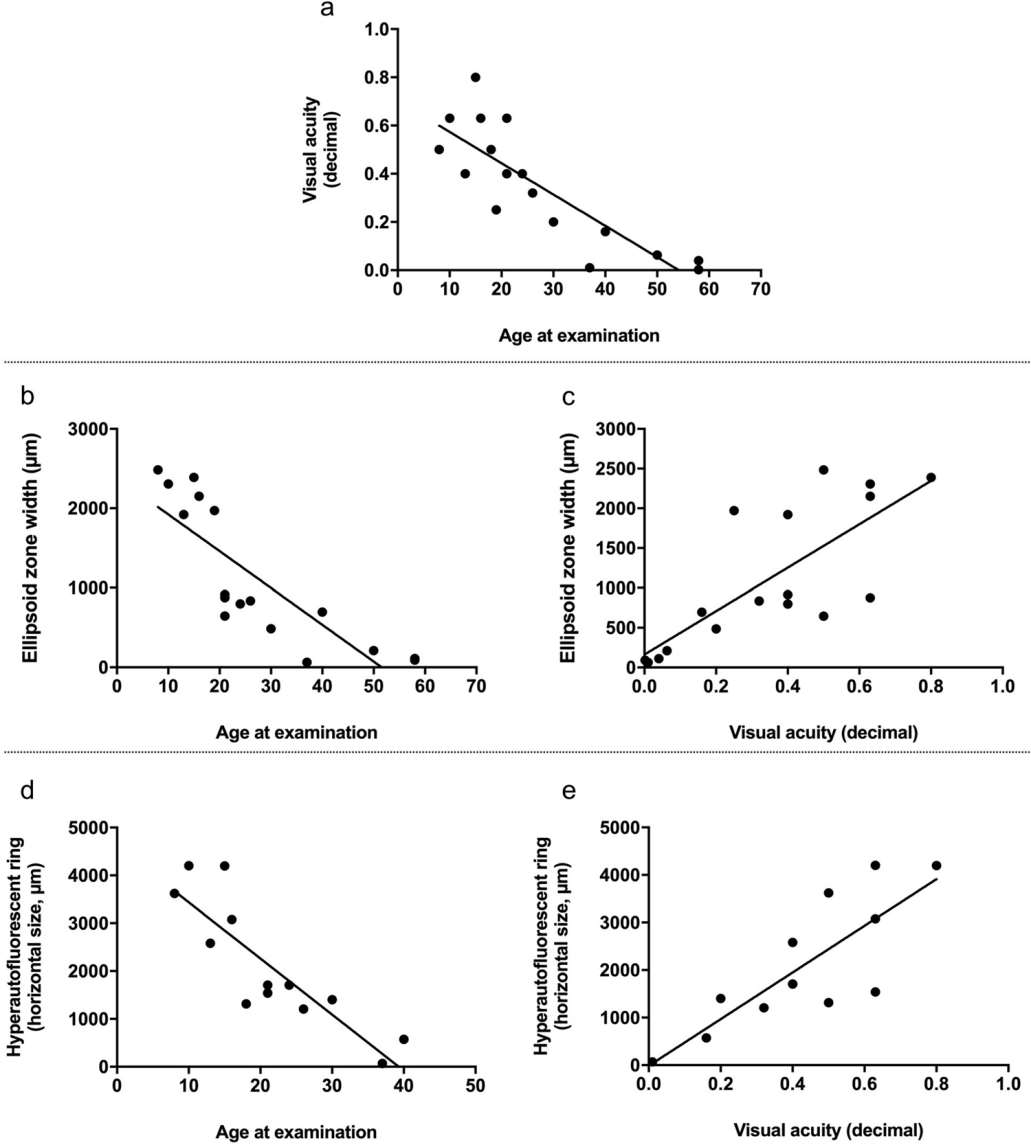
Morphologic parameters and visual acuity of patients with *RPGR*-associated retinitis pigmentosa. Age was negatively correlated with visual acuity (**a**), the length of the visible foveal ellipsoid zone (**b**), and the horizontal diameter of the hyperautofluorescent ring using short-wavelength autofluorescence (**d**). The ellipsoid zone width (**c**) and the hyperautofluorescent ring (**e**) were both positively correlated with visual acuity

RNFL thickness of the circumpapillary area was measured between the inner border of the internal limiting membrane and the inner layer of the ganglion cell layer using the integrated RNFL measurement tool of the HEYEX. In a first step, the software performed the automated RNFL detection. Subsequently, a human grader checked the results for accuracy and corrected the automated segmentation manually if needed. The manual segmentations were re-checked by another independent grader. While the automated RNFL segmentation worked well for all healthy controls, it could not accurately segment the RNFL in all patients with retinitis pigmentosa and had to be corrected manually. Whole retinal thickness was peripapillary measured and obtained after manual segmentation of the internal limiting membrane and the retinal pigment epithelium/Bruch’s membrane complex. Non-RNFL layers were calculated by subtracting the RNFL from the whole retinal thickness. Mean values are reported globally as well as for six sectors relative to the disc-fovea axis (temporal, temporal superior, temporal inferior, nasal, nasal superior, nasal inferior).

### Statistical analysis

Only the right eye of each patient was included. Statistical analysis was performed using GraphPad Prism v8.0 (GraphPad Software, La Jolla, CA, USA) and R [37]. The goodness-of-fit between the variables (BCVA, age, refraction, EZ, RNFL, refraction, hyperautofluorescent ring) was evaluated by (adjusted) *R*^2^ [38]. Multivariate regression with RNFL as dependent variable was performed accounting for potential interactions between the analyzed variables.

## Results

Seventeen consecutive and unrelated male patients with *RPGR*-associated RP were included in this study. In all patients, the clinical diagnosis of RP was established based on patients’ history, characteristic RP fundus features including bone-spicule-like hyperpigmentation, vascular attenuation, optic disc pallor, and severely reduced or extinguished amplitudes on ERG examination. Median age at first symptoms was 8 years (interquartile range [IQR], 3-10 years). Median age at study examination and retinal imaging was 21 years (IQR, 16-39 years) and median BCVA (decimals) was 0.4 (IQR, 0.1–0.6). Cross-sectional analysis showed a negative correlation of visual acuity with age (slope= − 0.013 ± 0.002 decimal units BCVA/year; *R*^2^ = 0.68) (Fig. 1a).

On OCT scans recorded along the horizontal meridian, the width of the visible ellipsoid zone was negatively correlated with age (slope = − 45.37 ± 8.14 µm/year; *R*^2^ = 0.65) (Fig. 1b). By visual inspection, this effect seemed more pronounced in patients below the age of 20 years than in older patients. The width of the ellipsoid zone was positively (*p* < 0.001) correlated with visual acuity (slope= 2729.1 ± 593.5 µm per decimal unit BCVA; *R*^2^ = 0.56; *p* < 0.001) (Fig. 1c).

A symmetrical macular hyperautofluorescent ring using AF imaging was identified in 13 out of the 17 (76%) patients, the other 4 patients revealed either diffuse (*n* = 2) or patchy chorioretinal (*n* = 2) alterations on AF imaging. Patients with a hyperautofluorescent ring were younger (median age 21 vs. 54 years; *p* = 0.003) and had a better visual acuity (median BCVA 0.4 vs. 0.063; *p* = 0.0004) than patients without a ring. The horizontal measured diameter of the ring was negatively correlated with age (117.32 µm/year; *R*^2^ = 0.72; *p* < 0.001) which seemed by visual inspection more pronounced in patients below the age of 20 years (Fig. 1d) and positively correlated (*p* = 0.03) with visual acuity (slope = 4909 ± 1062 µm per decimal unit BCVA; *R*^2^ = 0.63; *p* < 0.001) (Fig. 1e).

**Fig. 2:**
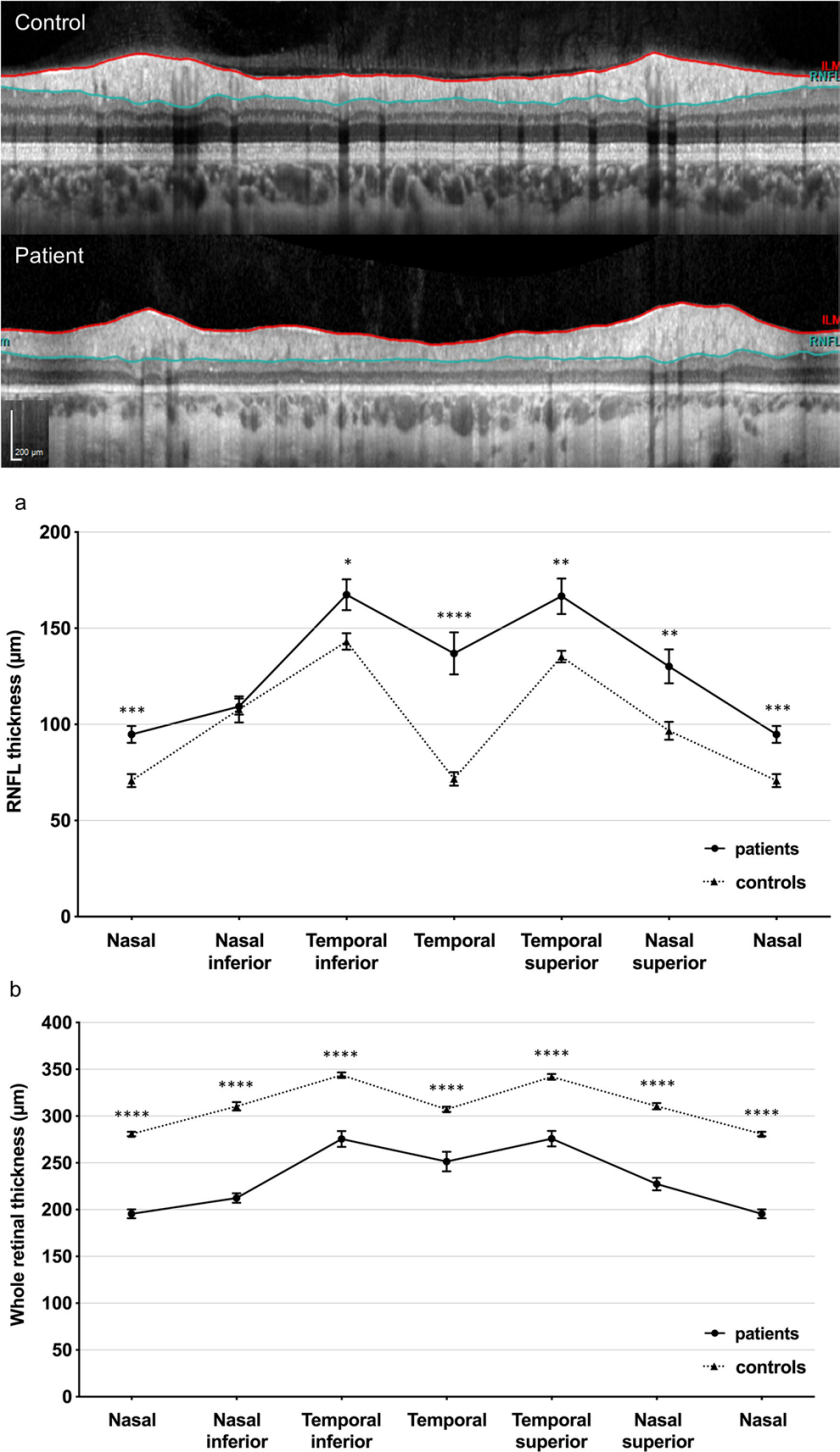
Representative peripapillary OCT scans of a healthy control and of a patient with *RPGR*-associated retinitis pigmentosa (top); sector-wise comparison of the retinal nerve fiber layer thickness (**a**) and whole retinal thickness (**b**) between *RPGR*-associated retinitis pigmentosa and controls. *p < 0.05, **p < 0.005, ***p < 0.0005, ****p < 0.0001

Measurement of mean global RNFL thickness in patients with *RPGR*-associated RP revealed significantly higher values compared to controls (128 µm vs. 96 µm; *p* < 0.0001). On a topographic level, the RNFL was significantly thickened in all sectors except for the nasal inferior sector (109.3 µm vs.107.8 µm) (Fig. 2a). The greatest thickness difference was seen in the temporal sector (136.9 µm vs.71.7 µm). In contrast, thinning of the outer retinal layers was observed in patients with *RPGR*-associated RP compared to controls. This resulted in significantly lower values of the whole retinal thickness in *RPGR*-associated RP globally (231 µm vs.309 µm; *p* < 0.0001) and in all different sectors (all, *p* < 0.0001) (Fig. 2b).

**Fig. 3:**
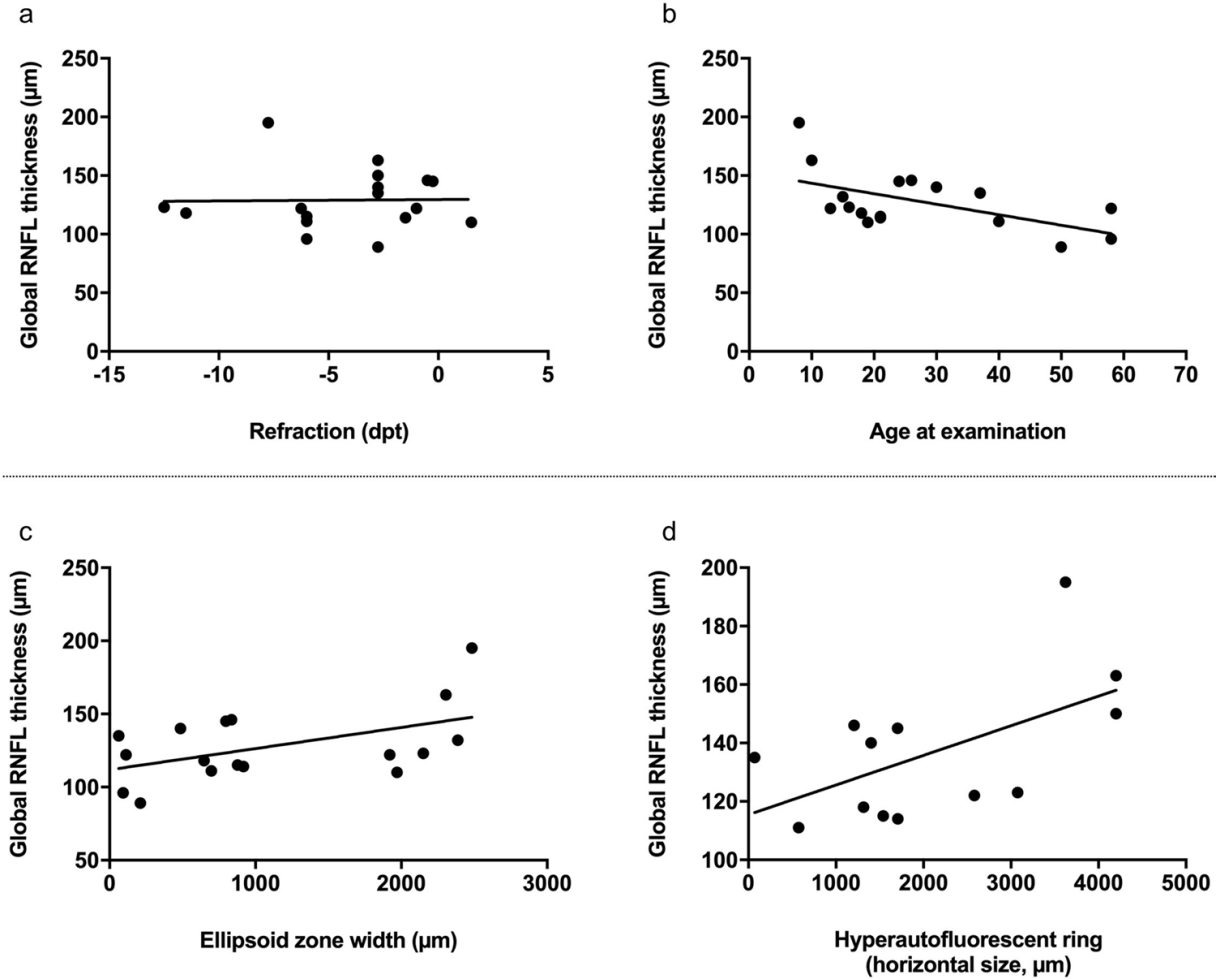
Comparison of the global retinal nerve fiber layer thickness of patients with *RPGR*-associated retinitis pigmentosa to refraction (**a**), age at examination (**b**), ellipsoid zone width (**c**), and the horizontal measured diameter of the hyperautofluorescent ring (**d**)

Subsequently, the impact of refractive error, age (both of which have been shown to affect RNFL thickness in normal eyes), EZ width, and the hyperautofluorescent ring diameter on RNFL thickness were analyzed. In univariate regression, age, the ellipsoid zone width and the hyperautofluorescent ring diameter were significantly associated with RNFL thickness (*p* = 0.014, *p* = 0.02, and *p* = 0.04, respectively) (Fig. 3b–d). However, no effect was observed for refractive error (*p* = 0.95) (Fig. 3a). Note that the EZ width and hyperautofluorescent ring diameter by themselves were highly correlated (Pearson’s correlation coefficient: 0.96).

To evaluate the individual contribution of each variable and account for possible interaction effects, multivariate linear regression was performed. After forward selection, age, EZ width and the interaction between those two were included in the final term (results provided in Supplementary Table 3). In summary, per year of age, the RNFL thickness decreased by 0.58 µm and per µm of EZ loss, the RNFL thickness decreased by 0.024 µm. Both factors alone, however, did not remain significant with the interaction term introduced into the model. Of note, with the limited number of *RPGR*-associated RP patients and the resulting moderate number of patients included in this analysis, the absence of statistical significance does not necessarily reflect the absence of a biological effect, but rather the limited power of the analysis. Finally, the interaction between age and ellipsoid zone width was significantly associated with RNFL thickness (*p* = 0.04): A patient who is 1 year older upon examination and has a 1 µm thinner EZ will have a 0.002 µm additional RNFL loss as compared to a patient where EZ remained unchanged over that year. Considering EZ loss as a surrogate for disease progression, these findings indicate that increased RNFL thickness was less pronounced in progressed disease. Together, the parameters included in this model explained 43.4% of the variability in RNFL thickness (i.e., adjusted *R*^2^ = 0.434).

## Discussion

Our cross-sectional structural-functional approach of patients with *RPGR*-associated RP revealed that visual acuity, the EZ width, and the diameter of the hyperautofluorescent ring negatively correlated with age. The correlation between age and EZ width, with larger EZs in younger patients and a pronounced EZ decline in patients under the age of 20 years, is in accordance with previous reports identifying a faster rate of EZ decline in younger patients regardless of the underlying *RPGR* variant [22, 24].

A ring of increased autofluorescence was observed in 76% of our patients, which is slightly higher than observed in previous studies [23, 39]. Patients with a ring were younger and exhibited a better visual acuity than patients without rings, indicating more advanced disease in patients without rings, which might also explain different frequencies of this feature across studies. Patients under the age of 20 years revealed a more pronounced reduction of the horizontal ring diameter compared to older patients, indicating a decline in progression rate with age, also seen in other cohorts of *RPGR*-associated RP [23].

Quantification of RNFL thickness in *RPGR*-associated RP revealed a global and section-wise RNFL thickening, more prominent in the temporal than in the nasal sections. While RNFL thickening was independent of refraction error, an otherwise known effector of RNFL thickness, a lower RNFL thickness in older RP patients was observed. The calculated RNFL thickness decrease (per year of age) was even more pronounced as compared to healthy controls [40]. Moreover, RNFL thickening was less pronounced in advanced disease, measured by EZ width or the diameter of the hyperautofluorescent ring as indicated by multivariant regression analysis. While our study of *RPGR*-associated RP identified RNFL thickening in all patients, future studies may investigate whether and to what extent differences in RNFL thickness are present between different molecular disease causes. This may also explain the variability of RNFL thinning and thickening previously observed in RP [28–34]. However, as manual segmentation was necessary for all our RP patients, it cannot be excluded that segmentation artifacts or different OCT devices also contributed to the RNFL differences between previous studies.

Causes for RNFL thickening in RP but also in patients with other inherited retinal diseases such as choroideremia are currently not well understood and various potential explanations have been considered [32, 41]. These include microglial remodeling secondary to outer retinal atrophy or altered metabolic signaling, blood vessel architecture of the inner retina, or yet unknown factors [41–45]. However, with a lack of histological data, explanations of RNFL thickening remain, to some degree, speculative at present [41].

Phenotypic characteristics, as seen in this study, are of importance for patient selection and outcome measurements in interventional trials, but also for patient counseling on the future disease course. Individual characteristics may have different importance for particular aspects. For example, examining a ring of increased autofluorescence appears adequate evaluating patients with less advanced disease, while the EZ width may also reflect progressed disease. As the status of the inner layer is, for instance, crucial for patients qualifying for gene replacement therapies, retinal implants, optogenetic approaches, or induced-pluripotent stem cells, these results suggest at least no pronounced atrophy of the RNFL in *RPGR*-associated RP. However, as RNFL measurements are significantly thickened, the overall structural or functional integrity cannot be judged based on this study. Thus, further investigations of RNFL alterations appear important.

As *RPGR*-associated RP is a rare condition, we are aware of the potentially limited statistical power of this study due to the low number of included patients which is a common challenge in rare diseases. Moreover, the lack of longitudinal observations, the cross-sectional approach, and further factors influencing the RNFL represent limitations. In respect of the rapid developments of therapeutic approaches, additional studies appear prudent to validate these OCT-based findings in different cohorts of *RPGR*-associated RP but also across different molecular disease causes, and to test whether RNFL alterations can contribute to monitoring retinal dystrophies.

In conclusion, this study describes structural parameters and visual acuity of patients with *RPGR*-associated retinopathy and provides novel insights into RNFL alterations seen in RP patients. These findings may be of interest for future therapeutic approaches such as gene replacement therapies.

## Supplementary Information

Below is the link to the electronic supplementary material.

Supplementary file1 (DOCX 365 KB)
