# Supplementary material for: Analysis of imaging biomarkers and retinal nerve fiber layer thickness in RPGR-associated retinitis pigmentosa
Source: Graefes Arch Clin Exp Ophthalmol. 2021 Jul 21;259(12):3597–604. doi: 10.1007/s00417-021-05233-w (PMC8589744; doi:10.1007/s00417-021-05233-w)
Supplement: Supplementary file 1 — Supplementary file1 (DOCX 365 KB) [file 417_2021_5233_MOESM1_ESM.docx]

Supplementary Table 1: Characteristics of patients and controls

|  | Age [years] at examination (IQR) | Visual acuity [decimal]  (IQR) | Refractive error [diopters]  (IQR) | Global RNFL thickness [µm] (IQR) | Ellipsoid zone width [µm]  (IQR) | Hyperautofluorescent ring [µm]  (IQR) |
| --- | --- | --- | --- | --- | --- | --- |
| *RPGR*-associated RP | 21 (16-39) | 0.4 (0.1-0.6) | -2.75 ([-6] - [-1.5]) | 128 (113-143) | 835 (485–1971) | 1705 (1315-3077) |
| Healthy controls | 21 (16-39) | 1 (1-1) | 0.25 (-0.25-0.75) | 96 (92-101) | not applicable | not applicable |
| p-value | 1 | <0.0001 | 0.0004 | p<0.0001 | not applicable | not applicable |

*RP = retinitis pigmentosa, IQR = interquartile range, RNFL = retinal nerve fiber layer*

Supplementary Table 2: *RPGR* variants

| ID | Gene | Zygosity | Exon/  Intron | Isoform | Nucleotide change | Protein change | Hg19 position | ACMG Classification |
| --- | --- | --- | --- | --- | --- | --- | --- | --- |
| 1 | *RPGR* | Hemizygous | ORF15 | NM_001034853.2 | c.2452G>T | p.Glu818* | chrX:38145800 | Pathogenic |
| 2 | *RPGR* | Heterozygous | ORF15 | NM_001034853.2 | c.2630del | p.Glu877Glyfs*212 | chrX:38145621 | Pathogenic |
| 3 | *RPGR* | Hemizygous | ORF15 | NM_001034853.2 | c.2405_2406del | p.Glu802Glyfs*32 | chrX:38145845 | Pathogenic |
| 4 | *RPGR* | Hemizygous | ORF15 | NM_001034853.2 | c.2236_2237del | p.Glu746Argfs*23 | chrX:38146014 | Pathogenic |
| 5 | *RPGR* | Hemizygous | ORF15 | NM_001034853.2 | c.2253_2260dup | p.Glu754Glyfs*64 | chrX:38145991 | Likely pathogenic |
| 6 | *RPGR* | Hemizygous | ORF15 | NM_001034853.2 | c.1991C>A | p.Ser664X | chrX:38146261 | Likely pathogenic |
| 7 | *RPGR* | Hemizygous | ORF15 | NM_001034853.2 | c.2253_2260dup | p.Glu754Glyfs*64 | chrX:38145991 | Likely pathogenic |
| 8 | *RPGR* | Hemizygous | ORF15 | NM_001034853.2 | c.2363_2364del | p.Glu788Glyfs*46 | chrX:38145887 | Likely pathogenic |
| 9 | *RPGR* | Hemizygous | ORF15 | NM_001034853.2 | c.2548del | p.Glu850Lysfs*239 | chrX:38145703 | Pathogenic |
| 10 | *RPGR* | Hemizygous | ORF15 | NM_001034853.2 | c.2939del | p.Glu980Glyfs*109 | chrX:38145312 | Likely pathogenic |
| 11 | *RPGR* | Hemizygous | ORF15 | NM_001034853.2 | c.2405_2406del | p.Glu802Glyfs*32 | chrX:38145845 | Pathogenic |
| 12 | *RPGR* | Hemizygous | ORF15 | NM_001034853.2 | c.2236_2237del | p.Glu746Argfs*23 | chrX:38146014 | Pathogenic |
| 13 | *RPGR* | Hemizygous | Exon 8 | NM_000328.3 | c.782del | p.Asn261Metfs*37 | chrX:38164039 | Likely pathogenic |
| 14 | *RPGR* | Hemizygous | Intron 13 | NM_000328.3 | c.1573-2A>G | splice cite | chrX:38147296 | Pathogenic |
| 15 | *RPGR* | Hemizygous | Exon 8 | NM_000328.3 | c.284G>A | p.Gly95Glu | chrX:38163905 | Pathogenic |
| 16 | *RPGR* | Hemizygous | Exon 4 | NM_000328.3 | c.917A>C | p.His306Pro | chrX:38180306 | Pathogenic |
| 17 | *RPGR* | Hemizygous | ORF15 | NM_001034853.2 | c.2442_2445del | p.Gly817Lysfs*2 | chrX:38145806 | Pathogenic |

Supplementary Table 3: Summary of multivariate regression analysis

| Variable | Slope | Std. error | Dimension | p-value |
| --- | --- | --- | --- | --- |
| Age | -0.580 | 0.53 | µm/y | 0.29 |
| Ellipsoid zone thickness | 0.024 | 0.01 | µm/µm | 0.07 |
| Interaction | -0.002 | <0.01 | µm(y*µm) | 0.04 |

Supplementary Figure 1: Representative patient images


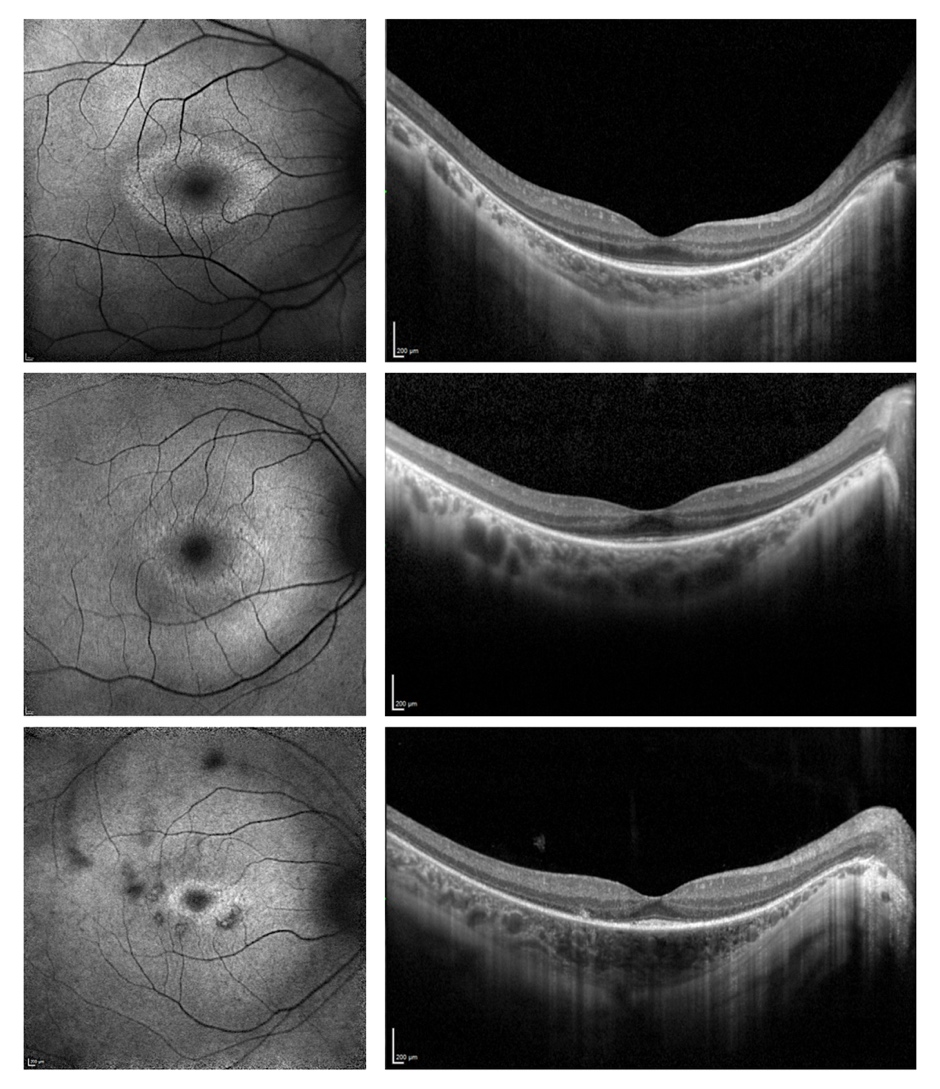


Exemplary fundus autofluorescence (left) and corresponding optical coherence tomography images (right) of patients with *RPGR*-associated retinitis pigmentosa and a macular hyperautofluorescent ring on fundus autofluorescence imaging. Visual acuity of the patients displayed was 20/32, 20/40 and 20/50, respectively.
